# Supplementary material for: Estimation of the heritability of medicinal compound contents in Glycyrrhiza uralensis
Source: PLoS One. 2025 Aug 18;20(8):e0327885. doi: 10.1371/journal.pone.0327885 (PMC12360535; doi:10.1371/journal.pone.0327885)
Supplement: S1 Fig — (1) A total of 240 seed-derived plants were cultivated over two years (2020–2021), and 31 clonal lines were established via stolon propagation. (2) Twenty-six clonal lines were cultivated for two years (2022–2023) using stolon-derived seedlings to estimate broad-sense heritability and analyze correlations among medicinal compound contents. (3) Thirteen clonal lines were cultivated for one year in both 2022 and 2023 to assess year-to-year variation. (4) Fourteen clonal lines were cultivated for one year in 2023 to compare one- and two-year-old plants. Stolon-derived seedlings were used in all experiments except in (1). Roots were sampled and analyzed for medicinal compound contents using HPLC. (PDF) [file pone.0327885.s001.pdf]

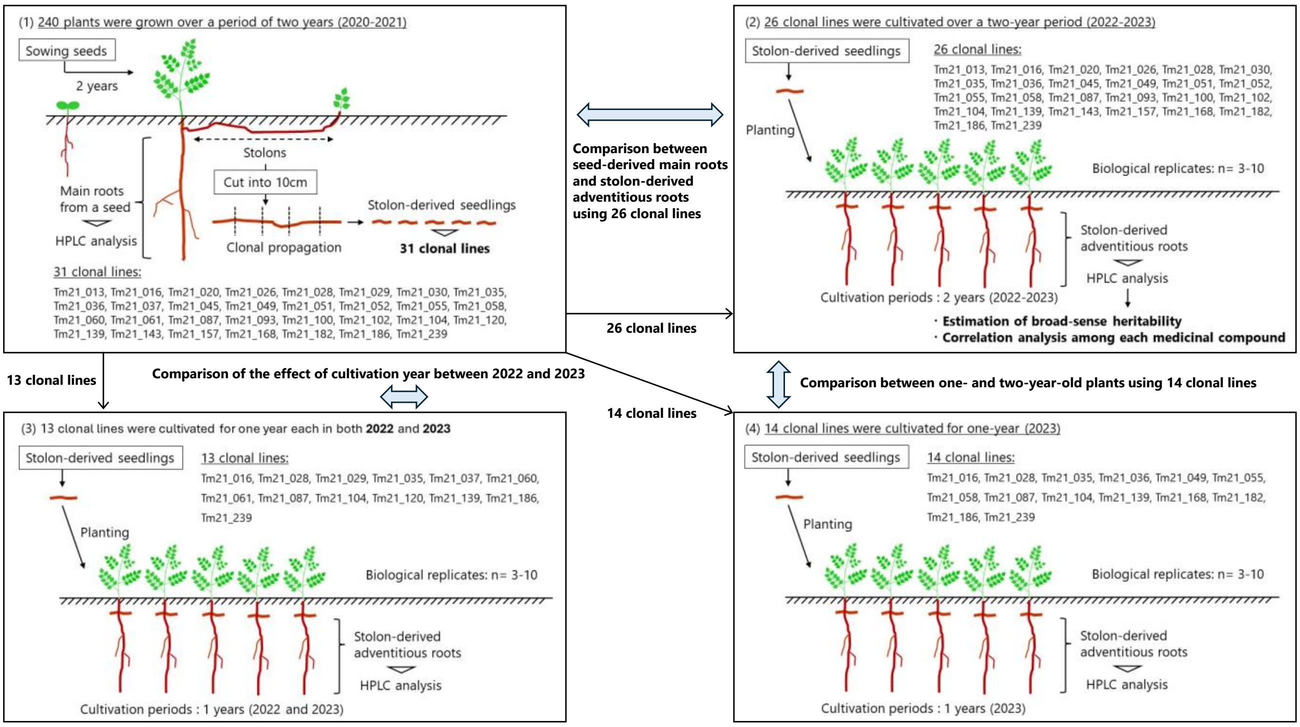

S1 Fig. Overview of the experimental design using clonal lines of *G. uralensis* to evaluate the heritability of medicinal compound contents. (1) A total of 240 seed-derived plants were cultivated over two years (2020–2021), and 31 clonal lines were established via stolon propagation. (2) Twenty-six clonal lines were cultivated for two years (2022–2023) using stolon-derived seedlings to estimate broad-sense heritability and analyze correlations among medicinal compound contents. (3) Thirteen clonal lines were cultivated for one year in both 2022 and 2023 to assess year-to-year variation. (4) Fourteen clonal lines were cultivated for one year in 2023 to compare one- and two-year-old plants. Stolon-derived seedlings were used in all experiments except in (1). Roots were sampled and analyzed for medicinal compound contents using HPLC.
