# Supplementary material for: Estimation of the heritability of medicinal compound contents in Glycyrrhiza uralensis
Source: PLoS One. 2025 Aug 18;20(8):e0327885. doi: 10.1371/journal.pone.0327885 (PMC12360535; doi:10.1371/journal.pone.0327885)
Supplement: S2 Fig — To facilitate comparison across compounds with different scales, all compound content values were standardized using Z-score normalization. Specifically, each value was transformed by subtracting the mean and dividing by the standard deviation for the corresponding compound, resulting in a distribution with a mean of 0 and a standard deviation of 1. Hierarchical clustering was subsequently performed on the standardized data to visualize phenotypic variation among the 31 clonal lines. Clustering was conducted based on Euclidean distance and Ward’s linkage method. (PDF) [file pone.0327885.s002.pdf]

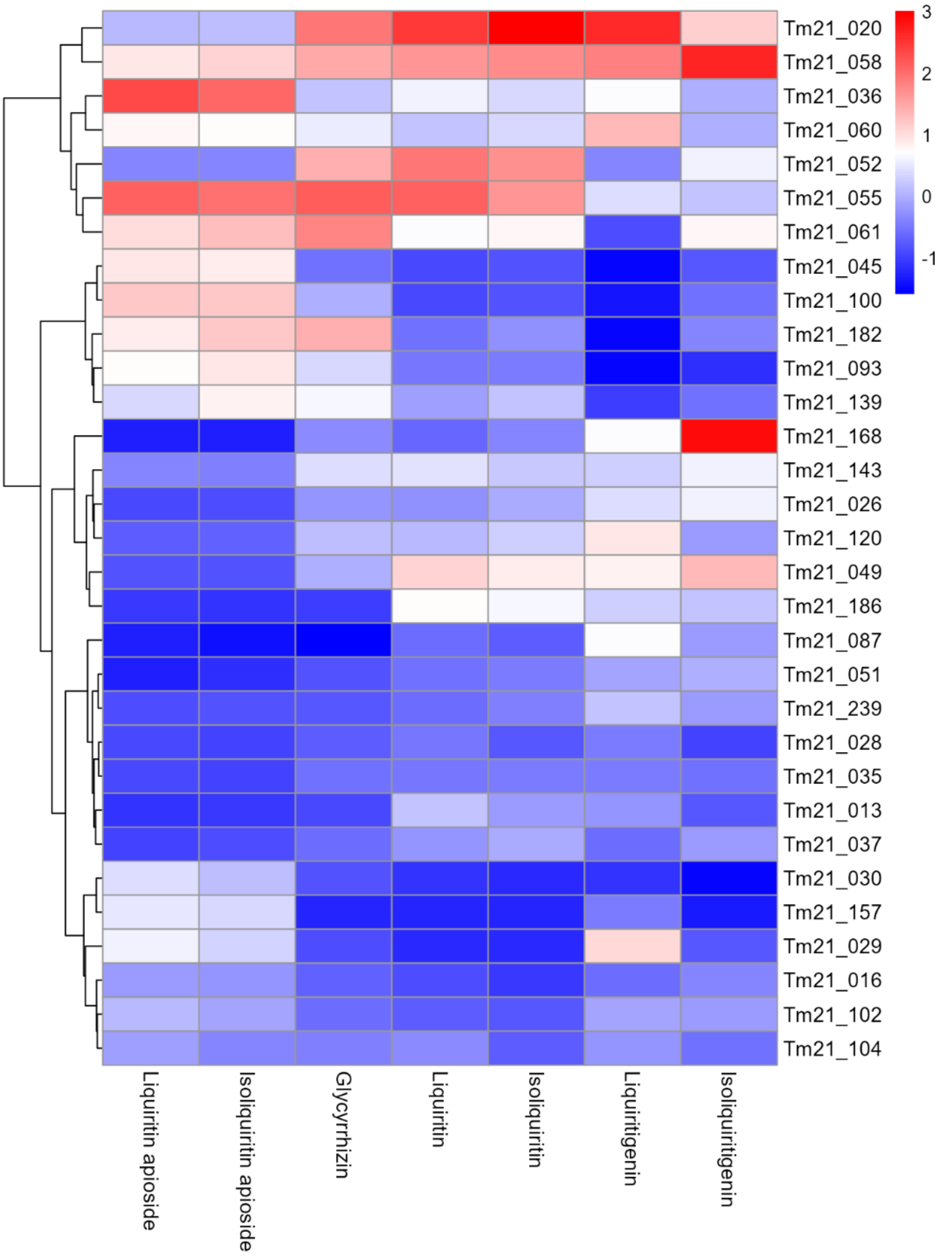

S2 Fig. Hierarchical clustering of 31 clonal lines based on standardized contents of seven medicinal compounds. To facilitate comparison across compounds with different scales, all compound content values were standardized using Z-score normalization. Specifically, each value was transformed by subtracting the mean and dividing by the standard deviation for the corresponding compound, resulting in a distribution with a mean of 0 and a standard deviation of 1. Hierarchical clustering was subsequently performed on the standardized data to visualize phenotypic variation among the 31 clonal lines. Clustering was conducted based on Euclidean distance and Ward's linkage method.
