# Supplementary material for: Estimation of the heritability of medicinal compound contents in Glycyrrhiza uralensis
Source: PLoS One. 2025 Aug 18;20(8):e0327885. doi: 10.1371/journal.pone.0327885 (PMC12360535; doi:10.1371/journal.pone.0327885)
Supplement: S1 Table — (PDF) [file pone.0327885.s003.pdf]

S1 Table. Climatic conditions around the research field where *G. uralensis* was cultivated in 2022 and 2023

	2022	2023
Cultivation period	2022/5/3-2022/11/18	2023/4/28-2023/11/20
Cultivation days	210	207
Average temperature (°C)	21.2	22.7
Accumulated mean temperature (°C)	4443.8	4689.1
Maximum temperature (°C)	38.1	38.1
Minimum temperature (°C)	4.0	3.6
Accumulated sunlight hours (h)	1109.1	1396.2
The number of days with temperatures above 35°C	59	85
Accumulated precipitation (mm)	655	940
