# Supplementary material for: Estimation of the heritability of medicinal compound contents in Glycyrrhiza uralensis
Source: PLoS One. 2025 Aug 18;20(8):e0327885. doi: 10.1371/journal.pone.0327885 (PMC12360535; doi:10.1371/journal.pone.0327885)
Supplement: Attachment — Submitted filename: Response to Reviewers.docx [file pone.0327885.s005.docx]

June, 18, 2025

*PLOS One*

Dear Reviewers

Thank you for the thoughtful and constructive feedback you provided regarding our manuscript. We agree with you and have incorporated these suggestions throughout our paper. Detailed responses on your suggestions are as follows:

**Reviewer #1’s comments**

Reviewer #1: The authors have carried quite detailed amount of work and carried out large experimentation on the genetic inheritance of the contents of seven medicinal compounds in *G. uralensis* and observed high heritability for the contents of glycyrrhizin, liquiritin, liquiritin apioside, isoliquiritin, and isoliquiritin apioside. This suggests that selective breeding could be an effective strategy for developing *G. uralensis* populations with high medicinal compound contents. *G. uralensis* cultivars with both high root yields and high medicinal compound contents could be developed and utilzed for commercial application. It is advised that kindly highlight the importance of these metabolites in the available herbal drugs or any other formulations used in their market so that in the future course of time, those newly breeding lines could be grown.

**Response to Reviewer #1**

Thank you very much for your constructive feedback on our manuscript. We fully agree with your suggestion. In response, we have added a description of the industrial importance of the medicinal compounds in lines 422–425 of the Discussion section.

**Reviewer #2’s comments**

Reviewer #2: This manuscript by Takahiro Tsusaka and Miki Sakurai described that they investigated the genetic inheritance (heritability) of medicinal compound contents in *G. uralensis* by analyzing clonal lines propagated from stolons. And they conclude that there is strong potential for selective breeding to enhance the medicinal quality of *G. uralensis* by targeting high-yielding genotypes with superior compound profiles and that this potential may also apply to the sustainable cultivation of other high-quality medicinal plant resources. The work is interesting. But there are still some parts need to be enhanced.

1. In the parts of plant materials and cultivation, the relationship among the materials used in the different experiments is not clear enough, especially when comparing the status of annual and biennial *G. uralensis* (Is the annual liquorice material a subset of the biennial? ), and could be supplemented with a designed figure or table to illustrate this issue?

2. In the parts of plant materials and cultivation, Please provide the latitude and longitude information of the experimental site.

3. Sample size varies considerably among the clonal lines, from 3 to 9, and it is recommended that the data be supplemented to a sample size of 5 or more.

4. Genetic analysis using clonal lines requires sufficient genetic distance among different lines. However, the 26 clonal lines in this study came from the same batch of seeds, and how about the genetic distance between them? So experimental evidence of how genetically distant they are from each other is requested.

5. The data for the primary root in Figure 4, ‘Correlation of primary root and adventitious root components’, were measured only once (n=1). It is recommended to add repeated measurements of primary roots or to justify the single measurement.

6. Figure 1 (compound structure) is not cited in the main text. It is recommended that it be mentioned in the methods or results section or that Figure 1 be removed from the text.

**Response to Reviewer #2**

Thank you very much for your valuable and insightful comments. We have carefully considered each point and revised the manuscript accordingly. Our detailed point-by-point responses are provided below.

Comment 1: In the parts of plant materials and cultivation, the relationship among the materials used in the different experiments is not clear enough, especially when comparing the status of annual and biennial *G. uralensis* (Is the annual liquorice material a subset of the biennial? ), and could be supplemented with a designed figure or table to illustrate this issue?

Response: We have added a schematic figure illustrating the experimental design and the plant materials used in each experiment, which is included in Appendix S1.

Comment 2: In the parts of plant materials and cultivation, Please provide the latitude and longitude information of the experimental site.

Response: We have added the latitude and longitude information of the experimental site in lines 107–108 of the Materials and Methods section.

Comment 3: Sample size varies considerably among the clonal lines, from 3 to 9, and it is recommended that the data be supplemented to a sample size of 5 or more.

Response: Due to the difficulty of cultivating *G. uralensis*, we were unable to maintain uniform replication numbers across all clonal lines. Since the plant requires several years to grow, it is not feasible to increase the number of samples at this stage of the study.

However, we recognize that a sample size of five or more is desirable to ensure adequate statistical power for variance analysis. Therefore, we re-estimated the broad-sense heritability values using only the 15 clonal lines with five or more replicates and compared the results to those obtained using all 26 lines with three or more replicates. The heritability estimates based on the 15 lines were as follows: glycyrrhizin 0.69, liquiritin 0.64, liquiritin apioside 0.86, liquiritigenin 0.29, isoliquiritin 0.66, isoliquiritin apioside 0.91, and isoliquiritigenin 0.15. These values were nearly identical to those obtained from the dataset of 26 clonal lines, indicating that the use of lines with three or more replicates still yields reliable results.

Accordingly, we have clearly stated this limitation in lines 418–422 of the Discussion section and explained that, based on this consideration, we chose to proceed with the analysis using clonal lines with three or more replicates.

Comment 4: Genetic analysis using clonal lines requires sufficient genetic distance among different lines. However, the 26 clonal lines in this study came from the same batch of seeds, and how about the genetic distance between them? So experimental evidence of how genetically distant they are from each other is requested.

Response: Since genotyping would require cultivating additional plants, which takes several years, it is difficult to include such data in the current study. To provide some support for the genetic diversity among the clonal lines, we have added a statement in lines 115–116 of the Materials and Methods section noting that the clonal lines were randomly selected from a population of 240 individuals. In addition, we have included Appendix S2, which presents phenotypic variation in compound contents and a clustering analysis based on these traits among the 31 clonal lines used in the experiments. We plan to conduct a genotypic analysis in a subsequent study and report the results in a future publication.

Comment 5: The data for the primary root in Figure 4, ‘Correlation of primary root and adventitious root components’, were measured only once (n=1). It is recommended to add repeated measurements of primary roots or to justify the single measurement.

Response: Due to the biological characteristics of *G. uralensis*, which is highly self-incompatible and genetically heterogeneous, it is not possible to obtain genetically identical plants from seeds. As a result, only one main root sample per clonal line was available for analysis, and increasing the number of replicates was not feasible. We have acknowledged this limitation and added a statement in lines 419–420 of the Discussion section to clarify this point.

Comment 6: Figure 1 (compound structure) is not cited in the main text. It is recommended that it be mentioned in the methods or results section or that Figure 1 be removed from the text.

Response: Thank you for pointing this out. We have added a reference to Figure 1 in lines 175–178 of the Results section to address this issue.

We look forward to hearing from you regarding our submission and would be happy to address any further questions or comments you may have.

Best regards.

Takahiro Tsusaka (TT), Miki Sakurai (MS)

1. Botanical Raw Materials Cultivation Technology Development Department, Tsumura & Co., Ami, Ibaraki, Japan (TT)

Phone: +812-9889-3833

Fax: +812-9889-2158

E-mail: [tsusaka_takahiro@mail.tsumura.co.jp](mailto:tsusaka_takahiro@mail.tsumura.co.jp) (TT)
